# *In Vitro* and *In Vivo* Characterization of the New Analgesic Combination Beta-Caryophyllene and Docosahexaenoic Acid 

**DOI:** 10.1155/2014/596312

**Published:** 2014-07-07

**Authors:** Paolo Fiorenzani, Stefania Lamponi, Agnese Magnani, Ilaria Ceccarelli, Anna Maria Aloisi

**Affiliations:** ^1^Department of Medicine, Surgery and Neuroscience, University of Siena, Via Aldo Moro, 53100 Siena, Italy; ^2^Department of Biotechnology, Chemistry and Pharmacy, University of Siena, Via Aldo Moro, 53100 Siena, Italy

## Abstract

Beta-caryophyllene (BCP) and docosahexaenoic acid (DHA) are components of several plants with documented anti-inflammatory and analgesic effects in animal pain models. In the present study, *in vitro* and *in vivo* tests were carried out to evaluate their effects, alone or in combination, during long-lasting administration in a model of persistent pain. IR spectra of the two compounds were obtained to determine their chemical stability and then *in vitro* toxicity was evaluated in fibroblasts and astrocytes. In the *in vivo* tests, the analgesic effects of BCP and BCP+DHA were determined in male rats subjected to a model of persistent recurrent pain (three repetitions of the formalin test once a week) to mimic recurrent pain. Both substances were administered *per os* in almond oil for 2 weeks. Gonadal hormones were determined at the end of the tests to evaluate treatment-induced effects on their levels. BCP changed fibroblast and astrocyte survival in a dose-dependent manner and the effect was counteracted by DHA coadministration. In the *in vivo* tests, pain responses were significantly decreased in the BCP and BCP+DHA groups with respect to OIL after 1 and 2 weeks of treatment. Estradiol and testosterone levels were increased only in the BCP group. In conclusion, BCP alone or at lower concentration in combination with DHA was efficacious in modulating pain, showing a clear analgesic activity.

## 1. Introduction 

Pain (especially chronic pain) has finally achieved worldwide acceptance as a disease (and not only a symptom) with huge personal and social costs. Epidemiological statistics are alarming: in Europe, it is estimated that one in four adults has a chronic pain condition; in the US, it is believed that at least 38 million adults suffer from chronic pain [1]. For this reason, many efforts have been made in scientific and administrative circles to achieve acceptable, definitive solutions to chronic pain. Nevertheless, patients too often remain inadequately treated. Moreover, the beneficial effects of pain killers are often accompanied by serious, long-lasting drug-induced side effects [2].

The discovery of specific cannabinoid receptors (CB1 and CB2) [3] and their endogenous ligands [4] has led to a better understanding of the pharmacological actions and use of cannabinoids as therapeutic agents in various pain conditions. CB1 receptors are widely distributed throughout the central and peripheral nervous systems, and activation of these receptors produces psychotropic effects [3, 4]. Instead, CB2 receptors are expressed mainly by immune cells, act in the periphery and do not show psychotropic effects [5–7]. When activated, CB2 receptors can affect the release of chemical messengers (i.e., cytokines by immune cells) and can modulate immune cell trafficking ([7–11] for reviews). In both clinical and preclinical studies, cannabis has been shown to have analgesic effects at central and peripheral levels [12, 13].

Cannabinoid receptor ligands such as beta-*caryophyllene* (BCP, a FDA-approved food additive) selectively bind to peripheral cannabinoid receptors (CB2) and act as full agonists [14]. BCP has been shown to have multiple functions. In particular, it had strong anti-inflammatory and analgesic effects in rats and mice [14–16] in a model of acute single administration.

The omega-3 PUFAs eicosapentaenoic acid (EPA) and docosahexaenoic acid (DHA) (found in salmon and seaweed) have well-known beneficial activities. After being enzymatically converted into bioactive autacoids, they have inflammation-resolving properties [17]. It was recently reported that deficiencies in these PUFAs are linked to chronic inflammatory disease [17]. Once converted to bioactive metabolites, EPA and DHA are needed to resolve the inflammation acting through resolvins, natural endogenous regulators of the immune system. For these reasons, a possible synergistic effect of the combination of BCP and DHA on the modulation of inflammatory pain responses was assessed in the present study. First, we determined if BCP and DHA would chemically interact when mixed together; second, we evaluated the* in vitro* acute toxicity of BCP and DHA alone and in combination at different concentrations; third, we assessed whether the administration of BCP or BCP plus DHA would affect pain behaviour in a model of persistent pain, the formalin test; fourth, we used the determination of gonadal hormones to evaluate other possible effects induced by long-lasting administration of these substances.

## 2. Methods and Materials 

### 2.1. Substances

**Figure 1:**
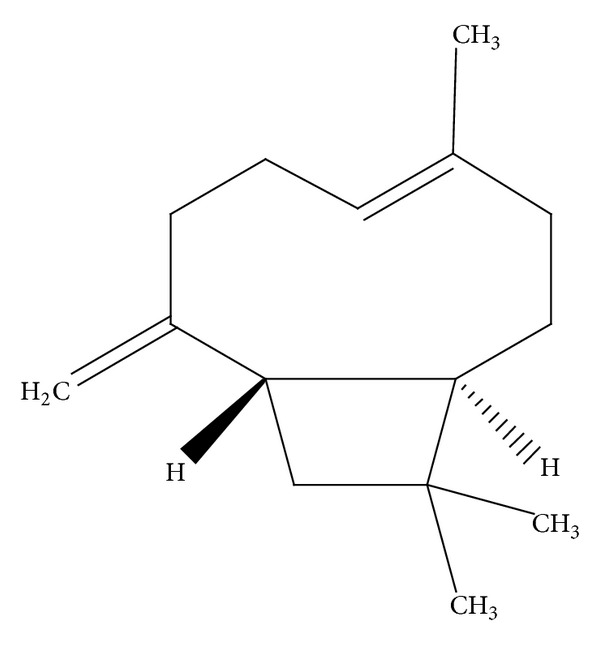


Beta-caryophyllene (BCP), a natural bicyclic sesquiterpene present in many essential oils, has the formula as in Figure 1.

**Figure 2:**
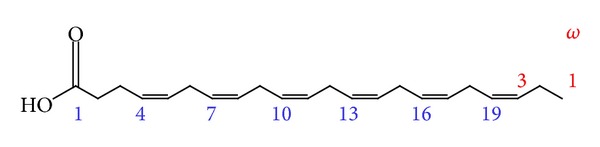


Docosahexaenoic acid (DHA), a long-chain essential fatty acid belonging to the omega-3 family, has the formula as in Figure 2.

### 2.2. Evaluation of Chemical Stability

The chemical stability of the two substances was analysed to determine if they would interact when mixed together. The IR spectra of BCP and DHA were obtained pure and in two mixtures with an FTIR Nicolet 5700-Thermo Spectrometer using an ATR (attenuated total reflectance) cell for liquids equipped with Ge crystal at 45°. The spectra (156 scans) were recorded at a resolution of 4 cm^−1^ using an MCT (mercury cadmium telluride) detector. Dry N_2_ was circulated in the cell to eliminate the CO_2_ and humidity in the air.

### 2.3. *In Vitro* Studies

#### 2.3.1. Materials for Cell Culture

Dulbecco's Modified Eagle's Medium (DMEM), trypsin solution, and all the solvents used for cell culture were purchased from Lonza (Belgium). Mouse immortalized NIH3T3 fibroblasts were obtained from American Type Culture Collection (USA). Human U373-MG astrocytes (deriving from a human astrocytoma line) were kindly provided by Professor Giampaolo Pessina (University of Siena).

#### 2.3.2. Procedures

Both cell lines were propagated in DMEM supplemented with foetal bovine serum at 10%, L-glutamine-streptomycin at 1%, and nonessential amino acids at 1% and incubated at 37°C in an atmosphere containing 5% CO_2_. At confluence, the cells were washed with PBS 0.1 M, detached from the support by means of a trypsin solution, centrifuged at 1500 rpm for 5 minutes at room temperature, and resuspended in complete medium (dilution 1 : 15). 15,000 cells suspended in complete medium were seeded in each well of a 24-well multiwell; the medium volume of each well was brought to 1 mL and the cells were left in an incubator at 37°C and 5% CO_2_ for 24 hours. At the end of incubation, dilutions of BCP and DHA were added to each sample and the multiwells were left to incubate for a further 24 hours at 37°C and 5% CO_2_. Each dilution of each sample was tested in 8 replications.

#### 2.3.3. Evaluation of Acute Toxicity

Toxicity was evaluated by determining the cell viability after 24 hours of incubation with BCP and DHA with the neutral red uptake (NRU) method according to the procedure of the National Toxicology Program (NTP) Interagency Center for the Evaluation of Alternative Toxicological Methods (NICEATM). The concentrations of BCP tested with both cell types, alone or mixed with DHA, were 1 × 10^−2^,  1 × 10^−4^,  1 × 10^−6^,  1 × 10^−8^, 1 × 10^−10^ and 1 × 10^−12^ M. The concentrations of DHA, used alone or in combination with BCP, were 3.5, 3.5 × 10^−2^, 3.5 × 10^−4^, 3.5 × 10^−6^, 3.5 × 10^−8^, and 3.5 × 10^−10^%.

First, the following solutions were prepared:

1. stock solution of neutral red: 0.33 g of neutral red in 100 mL of sterile distilled water;
2. medium with neutral red: 1.0 mL of stock solution + 99.0 mL of complete culture medium;
3. neutral red extraction solution: glacial acetic acid at 1% + ethanol at 50% + distilled water at 49%.

At the end of the incubation period, the culture medium was removed from each well and the cells were gently washed with 1 mL of PBS preheated to 37°C. The washing solution was removed and 1.0 mL of medium with neutral red was added to each well. The multiwells were placed in an incubator for 3 hours. At the end of the incubation time, the medium with neutral red was removed, each well was washed with 1 mL of PBS preheated to 37°C and exactly 1 mL of extraction solution was added to each well. At this point, the plates were placed on a shaker for 20–45 minutes at room temperature in order to extract the dye from the vital cells. During this phase, the plates were covered to protect them from light. Five minutes after the end of the shaking, the extraction solution was removed from each well and the absorbance was read at 540 nm in a UV/Vis spectrophotometer (Lambda 25, Perkin Elmer).

### 2.4. *In Vivo* Study

The* in vitro* acute toxicity study showed that BCP was toxic at high concentration, although its toxicity was completely reversed by concomitant DHA administration. Therefore, it was necessary to evaluate if the* in vivo* BCP analgesic activity would be maintained at a lower dose with the collaboration of a “safe” compound like DHA. The following groups were used to study the behavioural and hormonal responses to long-lasting treatment with BCP alone or in combination with DHA: group BCP, receiving BCP alone (5 mg/Kg/day), and group BCP+DHA, receiving half of the dose of BCP (2.6 mg/Kg/day, 52%) plus DHA (2.4 mg/Kg/day, 48%), with the OIL group receiving sweet almond oil as control. These doses were chosen following suggestions in the literature [14, 18, 19].

#### 2.4.1. Subjects

Twenty-four male Wistar Han rats were used (225 gr upon arrival). Throughout the experiment, the rats were kept in groups (4 per cage) with* ad libitum* access to food and water on a 12 h light/dark cycle with lights on at 7 pm. The experimental phases started one week after the arrival of the animals to allow them to habituate. All experimental procedures were carried out during the active phase of the animal's circadian cycle (10.00–12.00 am) in dedicated rooms provided with red light and white noise. The regulations of the European Communities Council Directive 86/609/EEC and the ethical guidelines for the study of experimental pain in conscious animals [20] were followed in all experimental phases. Permission from the Italian Ministry of Health (Ministero della Sanità) was obtained.

#### 2.4.2. Experimental Procedures

**Figure 3:**
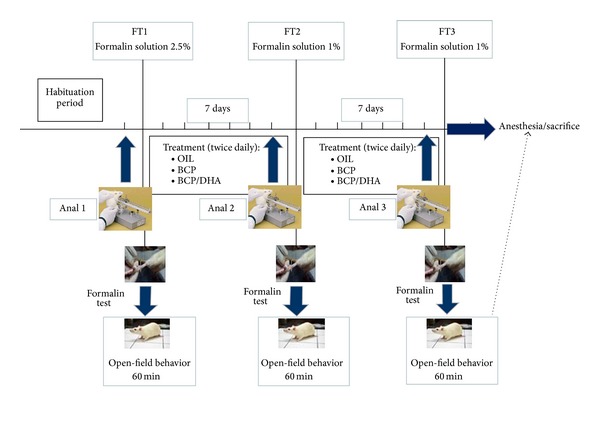
Schematic representation of the experimental design. See Section 2 for details.

As described in Figure 3, the animals were subjected to the analgesimeter (Anal 1) and formalin (FT1) tests before the beginning of substance treatment to evaluate the basal response of each one to the different experimental procedures. One week and two weeks after daily administration of the test substances, the animals were subjected to the second and third analgesimeter and formalin tests (Analg 2, FT2, Analg 3, and FT3, resp.). Immediately after the last formalin test (FT3), all animals were sacrificed.

*Analgesimeter Test*. This test was carried out to evaluate the phasic, mechanical paw-withdrawal threshold of awake, lightly restrained rats. The apparatus (Analgesimeter, Ugo Basile, Italy) applies a linearly increasing force to the dorsum of the rat's hind paw, resulting in the nociceptive flexion reflex. Each test consisted in two measurements in the right and left paws, with 15 minutes in between. They were carried out the day before the formalin test. The latency to paw-withdrawal was recorded and subsequently analysed. 

*Formalin Test*. On the day of the experiment, the animals were transported to the experimental room and gently restrained and injected with formalin (FT), after which they were immediately placed in the open-field apparatus where their behaviour was videotaped for 60 min for subsequent analysis. The first treatment with formalin (FT1, 50 *μ*L, s.c. in the dorsal right hind paw) was carried out at a concentration of 2.5% to induce a moderate but persistent inflammatory state. The second (FT2) and third (FT3) formalin treatments involved injection of the same amount of formalin (50 *μ*L) but at a very low concentration (1%) merely to induce spontaneous pain responses. This procedure was chosen because of the strong similarity with clinical pain episodes (recurrent pain). The formalin test was selected as the most relevant test to assess pain intensity in unrestrained animals. Indeed formalin injection induces a series of specific behaviour that can easily be quantified: licking and tonic flexing of the injected paw (measured in seconds) and paw jerk (phasic flexion, measured as frequency). Nonpain-related behaviour was also recorded as an index of motor activity: number of seconds spent exploring the environment (activity) and cleaning the body (self-grooming), number of times the animal reared to explore the environment (rearing), and time spent sitting alert or in sleeping-like posture (crouch).

At the end of each formalin test, the animals were returned to their home cages.

After the first formalin test, all animals belonging to the same cage were randomly assigned to the experimental groups: group OIL (*n* = 8), group BCP (*n* = 8), and group BCP+DHA (*n* = 8). The animals were treated by oral gavage for 15 days (twice a day) until the end of the experimental phases.

For increased objectivity and consistency, the behavioural analysis was carried out by a trained technician blind to the treatments.

*Hormone Determination*. Immediately after the third formalin test (FT3), the animals were anaesthetized, the abdomen was opened, and blood was collected from the abdominal vein in heparin-added syringes. Blood was centrifuged (3000 g for 10 min at 4°C) to obtain plasma and the samples were frozen at −20°C until the assay. To determine possible endocrine modulation by the treatments, we measured total testosterone and estradiol plasma levels with the ADVIA Centaur T assay, a competitive immunoassay using direct chemiluminescent technology. The intra-assay and interassay coefficients of variation were 5% and 8%, respectively, for all determinations.

*Data Analysis*. The acute toxicity data were analysed by one-way ANOVA with the factor Concentration (7 levels: control and 6 scalar doses of BCP, DHA, and BCP+DHA).

The analgesimeter latencies were expressed as percentage of variation recorded during the second and third tests with respect to the first one, separately for the right and left paws; analyses was carried out by one-way ANOVA with the factor Treatment (3 levels: OIL, BCP, and BCP+DHA).

All behavioural data were analysed by ANOVA with the factors Treatment (3 levels: OIl, BCP, and BCP+DHA) and Time (60 min, split into 12 periods of 5 minutes each) for each formalin test. Moreover, to evaluate the modulation of pain behaviour by treatments during the three formalin tests, ANOVA was applied to the total of pain behaviour recorded during the second phase (20–45 min) with the factors Treatment (3 levels: OIL, BCP, and BCP+DHA) and Test (3 levels: FT1, FT2, and FT3). Hormonal data were analysed by one-way ANOVA with the factor Treatment (3 levels: OIL, BCP, and BCP+DHA). The LSD post hoc test was applied when appropriate. The criterion for statistical significance was *P* < 0.05.

## 3. Results

### 3.1. Chemical Stability

**Figure 4:**
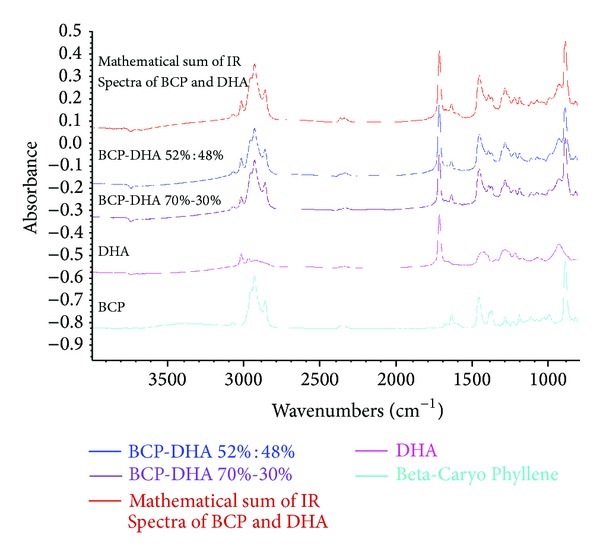
IR spectra of (from top to bottom) pure BCP, pure DHA, mixture BCP70%-DHA30%, BCP52%-DHA48%, and mathematical sum of the IR spectra of the two pure components (BCP and DHA).

As shown in Figure 4, the IR spectra of the two mixtures (BCP52%-DHA48% and BCP70%-DHA30%) were exactly superimposable on the spectrum obtained from the mathematical sum of the IR spectra of the two pure components (BCD spectrum + DHA spectrum in a ratio of 1 : 1). The infrared spectroscopy data allowed us to rule out the formation of a BCP-DHA complex with its own chemical identity. Hence, the structure and consequently the biological activity of the two single compounds in the mixture were preserved, since they were not compromised by any strong interaction involving the functional groups of the two molecules.

### 3.2. Acute Toxicity

The* in vitro* acute toxicity of the following compounds, as a function of their concentration, was tested: BCP, DHA, and BCP+DHA mixture (52%–48%).

**Figure 5:**
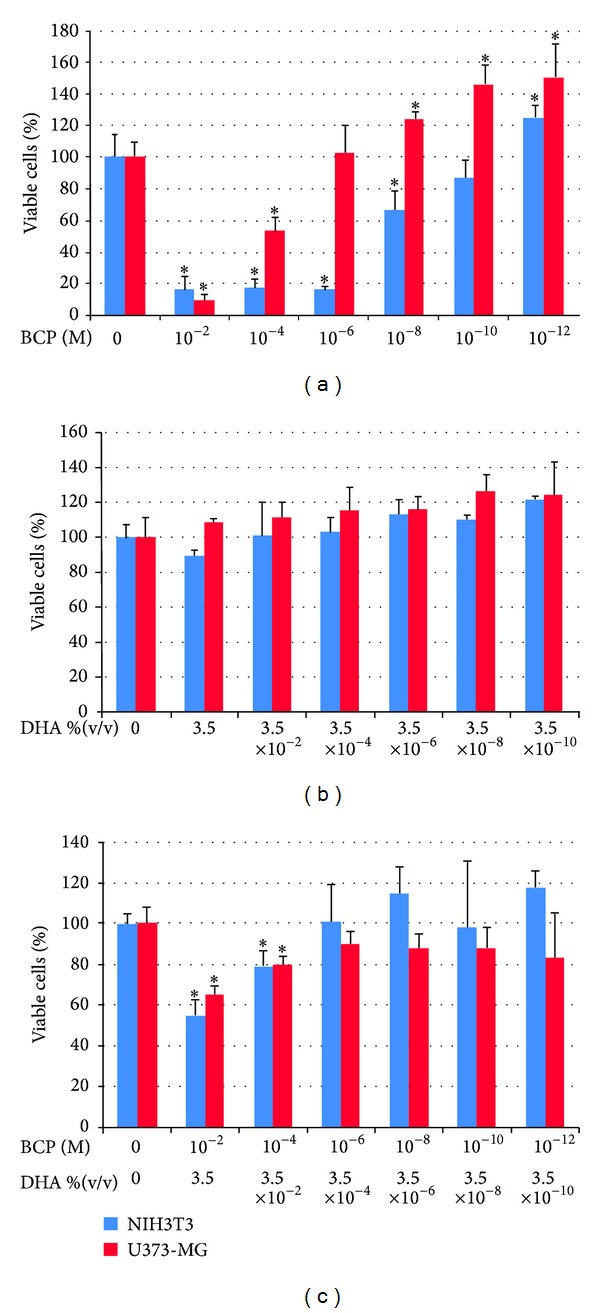
Viability of fibroblasts (NIH3T3) and astrocytes (U373-MG) after 24 hours of contact with different concentrations of BCP (a), DHA (b), or the BCP52%-DHA48% mixture (c). Data are mean ± SD of three experiments run in six replicates. *P < 0.05 versus control.

The graphs of cell vitality of NIH3T3 fibroblasts and U373 astrocytes in contact with the different concentrations of BCP, BCP+DHA, and DHA are given below. At the higher concentrations, BCP showed significant toxic effects, as evident from the lower vitality level in those preparations (Figure 5(a)). The effect was greater on the fibroblasts than on the astrocytes. For the astrocytes, it is interesting that BCP stimulated cell proliferation at a concentration lower than 1.0 × 10^−6^ M and that this activity increased with decreasing concentrations. All toxic effects disappeared in the BCP+DHA mixture (Figure 5(c)). DHA did not have a toxic effect on either the fibroblasts or the astrocytes at any of the concentrations (Figure 5(b)). Hence, the disappearance of the toxic effect of BCP can be attributed to the simultaneous presence in solution of DHA, although the presence of DHA also seemed to decrease the BCP stimulation of astrocyte proliferation (this occurred starting from a BCP concentration of 1.0 × 10^−6^ M, as shown in Figure 5(c)).

### 3.3. *In Vivo* Study

The animals showed no sign of discomfort during any of the experimental phases. Food was consumed regularly and body weight increased similarly in all groups. The* per os* procedure was carried out easily in all groups.

#### 3.3.1. Analgesimeter Test

**Figure 6:**
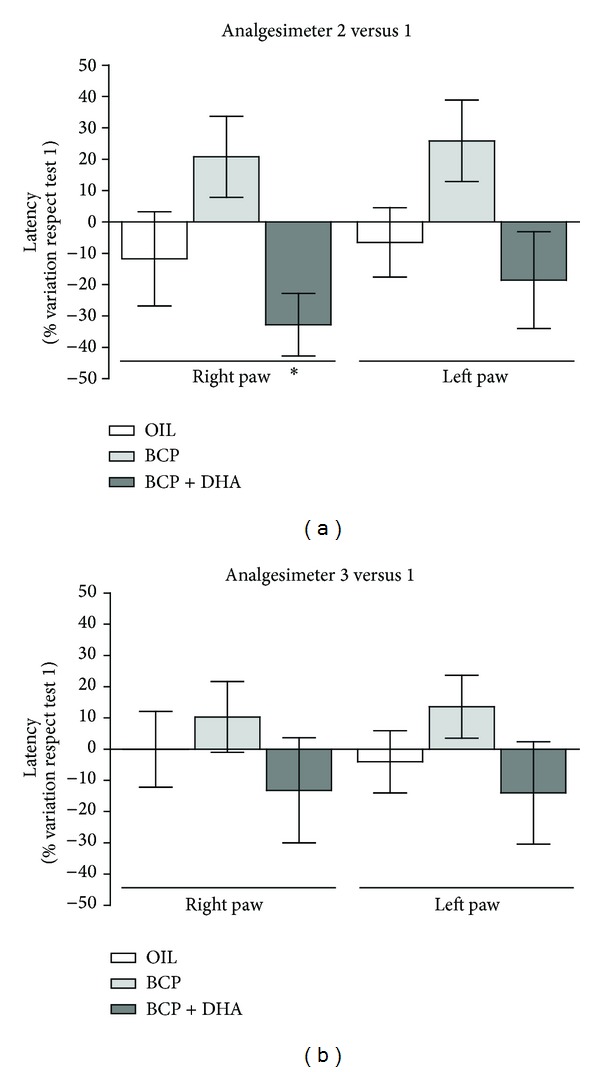
Analgesimeter test: histogram of the % variation of latency of paw-withdrawal recorded in analgesimeter tests 2 (a) and 3 (b) with respect to the mean latency recorded during the first test, in the right and left paw in animals treated with OIL (white), BCP (light gray), and BCP+DHA (dark gray). Values are means ± SEM. *P < 0.01 versus BCP.

This test was carried out to evaluate the effect of one and two weeks of BCP and BCP+DHA treatment on the mechanical paw-withdrawal threshold with respect to basal conditions (Analg 1). One-way ANOVA applied to the percentage of variation of latency recorded during the second and third tests with respect to the first one revealed different effects of the treatments, while BCP alone always resulted in higher values, that is, analgesia, BCP+DHA tended to produce lower (hyperalgesic) levels. In particular, there was a significant effect of treatment during the second test (Analg 2) for the right (treated) paw (*F*_2,17_ = 4.25; *P* < 0.03) due to the significant difference between the BCP and BCP+DHA groups (Figure 6).

#### 3.3.2. Formalin Test

During the formalin test, both spontaneous behaviour and formalin-induced responses were measured and quantified for comparisons.

*Spontaneous Behaviour*. ANOVA applied to the spontaneous behaviour and body weight recorded along the experimental sessions did not show significant differences among groups; hence, the results are not shown. 

*Pain Responses*. When treated with formalin and placed in the experimental apparatus, all animals showed characteristic behavioural responses present in the formalin test: licking, flexing, and paw jerk. As expected, these responses showed a biphasic time course characterized by an initial burst of pain behaviour (first phase, 0–5 min) followed by an intermediate phase with a drastic decrease of all pain responses (interphase, 5–20 min) and the second phase (20–60 min) in which inflammatory events certainly play the major role.

*Analysis of the Differences among Groups in Each Test*

*Formalin Test 1 (FT1)*. During this test, data were collected only to evaluate the effect of treatment with respect to basal conditions. After this test, animals belonging to the same cage were randomly assigned to one of the three experimental groups. 

*Formalin Test 2 (FT2)*

**Figure 7:**
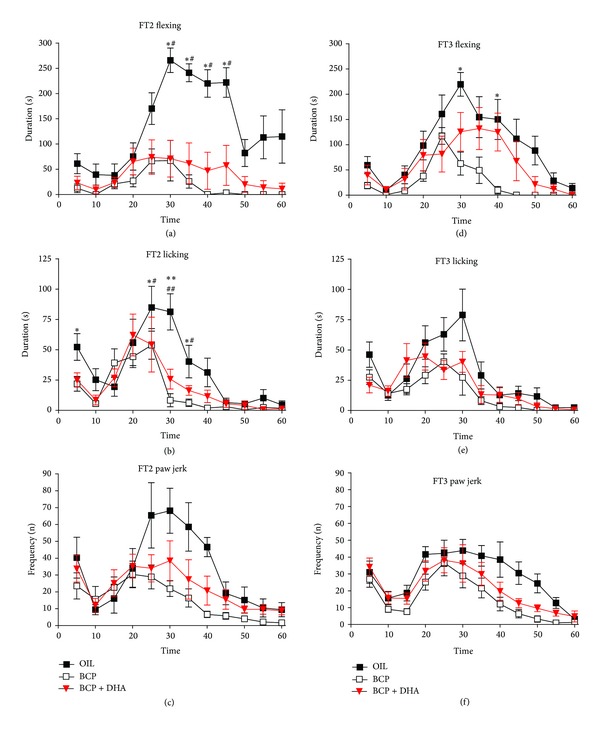
Formalin test: time course of formalin-induced flexing (AD), licking (BE), and jerking (CF) of the injected paw during FT2 and FT3 in the OIL (■), BCP (□), and BCP+DHA (▼) groups. ANOVA showed that BCP and BCP+DHA administration significantly reduced the formalin-induced responses, in particular, during FT2. Values are means ± SEM. See Section 3 for details. **P < 0.001 versus BCP, *P < 0.05 versus BCP, ^##^ *P* < 0.001 versus BCP+DHA, and ^#^ *P* < 0.05 versus BCP+DHA.

*Flexing*. One-way ANOVA applied to flexing duration revealed a significant effect of Treatment (*F*_2,220_ = 18.26; *P* < 0.001) due to the significantly lower levels in the BCP and BCP+DHA groups (*P* < 0.0001 for both) with respect to the OIL group. In particular, the interaction Treatment × Time (*F*_22,220_ = 3.8; *P* < 0.001) showed that the reduction of the formalin-induced flexing of the paw was due to the lower levels from 30 to 45 min of FT2 with respect to OIL-treated animals (*P* < 0.01 for BCP and *P* < 0.05 for BCP+DHA) (Figure 7(a)). 

*Licking*. One-way ANOVA also showed a significant effect of Treatment on licking duration (*F*_2,220_ = 11.19; *P* < 0.001) due to the lower levels in the BCP and BCP+DHA groups than in the OIL group (*P* < 0.001 and *P* < 0.002, resp.). In particular, the Treatment × Time interaction (*F*_22,220_ = 1.78; *P* < 0.019) showed that significant differences between the OIL and the treated groups were already present during the first phase and the early second phase (20–35 min), as shown in Figure 7(b). 

*Paw Jerk*. ANOVA applied to paw jerk frequency revealed a significant effect of Treatment (*F*_2,220_ = 9.12; *P* < 0.001) due to lower levels in the BCP group than in the OIL group (*P* < 0.001). In particular, the Treatment × Time interaction (*F*_22,220_ = 1.57; *P* < 0.05) showed a reduction of formalin-induced jerking of the paw by both substances during the second phase (in the 30–35 min interval) of FT2 with respect to OIL-treated animals (*P* < 0.05 for both treatments) (Figure 7(c)). 

*Formalin Test 3 (FT3)*

*Flexing*. ANOVA showed a significant effect of Treatment on flexing duration (*F*_2,220_ = 5.896; *P* < 0.009) due to the lower levels in the BCP group than in the OIL group (*P* < 0.002). Moreover, as shown in Figure 7(d), the significant Treatment × Time interaction (*F*_22,220_ = 2.12; *P* < 0.003) was due to a reduction of formalin-induced flexing in the BCP group in the 30–40 min interval of the test with respect to OIL-treated animals (*P* < 0.05 for both intervals). 

*Licking*. ANOVA revealed a significant effect of Treatment on licking (*F*_2,220_ = 11.12; *P* < 0.0005) due to lower levels in BCP and BCP+DHA animals than in the OIL group (*P* < 0.001 and *P* < 0.001, resp.) (Figure 7(e)).

*Paw Jerk*. ANOVA applied to paw jerk frequency showed a significant effect of Treatment (*F*_2,220_ = 4.73; *P* < 0.02) due to the lower levels in the BCP group than in the OIL group (*P* < 0.005) (Figure 7(f)). 

*Analysis of the Differences within Groups in the Three Tests*. To evaluate the effects of treatments on the modulation of pain responses during the repeated formalin tests, ANOVA was applied to flexing, licking, and paw jerk recorded during FT1, FT2, and FT3 with the factors Group and Test. The cumulative sum of each behavioural response during the early second phase (20–45 min) was chosen as the most representative of the treatment effects. 

**Figure 8:**
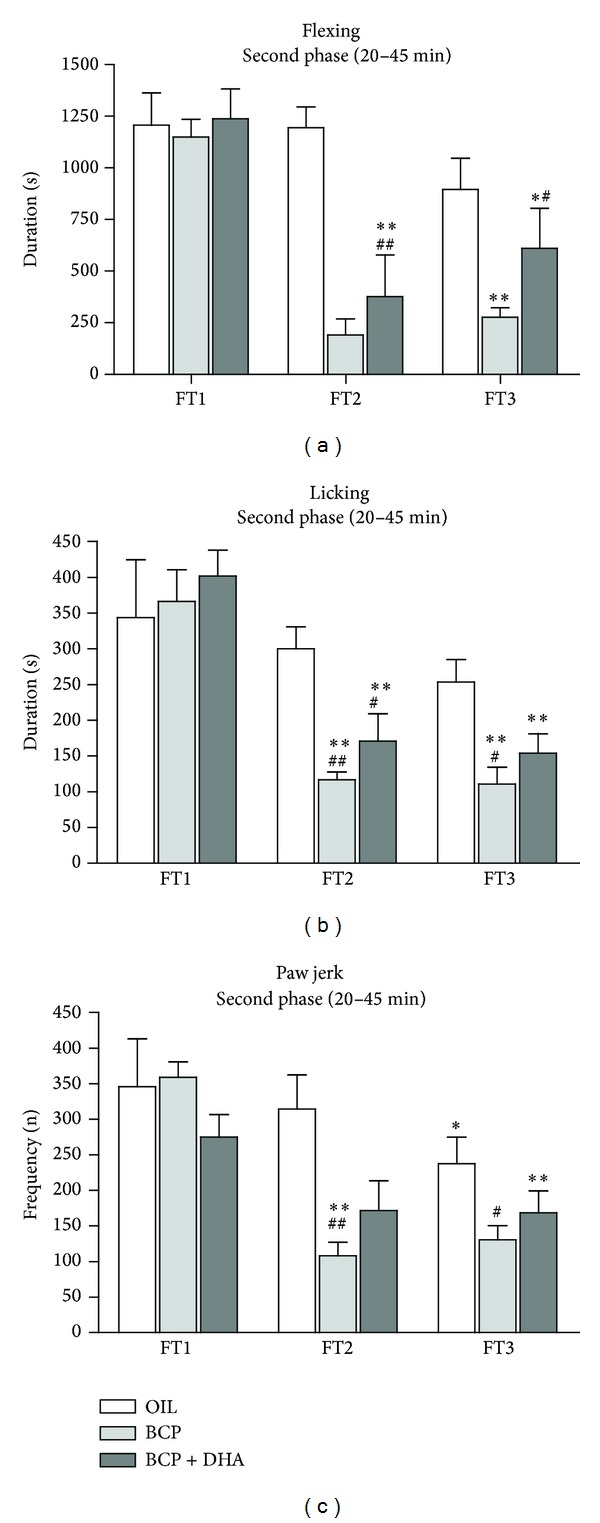
Flexing (a), licking (b), and jerking (c) of the injected paw in OIL (white), BCP (light gray), and BCP+DHA (dark gray) groups during the second phase (20–45 min, a–c) of FT1, FT2, and FT3. ANOVA showed that BCP and BCP+DHA administration significantly reduced the formalin-induced responses, in particular, during the second phase of FT2 and FT3. Values are means ± SEM. **P < 0.0001 versus FT1; *P < 0.02 versus FT1; ^##^ *P* < 0.0001 versus OIL, same test; ^#^ *P* < 0.01 versus OIL, same test.

*Flexing.* Two-way ANOVA revealed a significant effect of Treatment (*F*_2,62_ = 12.25; *P* < 0.001), Test (*F*_2,62_ = 18.92; *P* < 0.001), and the Treatment × Test interaction (*F*_4,62_ = 3.63; *P* < 0.01). As shown in Figure 8(a), this was due to the reduction of formalin-induced flexing in the BCP group in FT2 and FT3 with respect to FT1 (*P* < 0.001 for both). In the BCP+DHA group, flexing duration was decreased in FT2 and FT3 with respect to FT1 (*P* < 0.0001 and *P* < 0.002, resp.) but was lower than in the OIL group only during FT3 (*P* < 0.002). 

*Licking.* Two-way ANOVA showed only a significant effect of Treatment (*F*_2,62_ = 4.53; *P* < 0.014) due to the lower levels in the BCP group than in the OIL group (*P* < 0.005) (Figure 8(b)). 

*Paw Jerk*. Two-way ANOVA revealed a significant effect of Treatment (*F*_2,62_ = 6.34; *P* < 0.003), due to the lower levels in the BCP and BCP+DHA groups (*P* < 0.003 for both) and of Test (*F*_2,62_ = 13.05; *P* < 0.001) due to the lower levels in FT2 and FT3 with respect to FT1 (*P* < 0.001 for both) (Figure 8(c)). 

**Figure 9:**
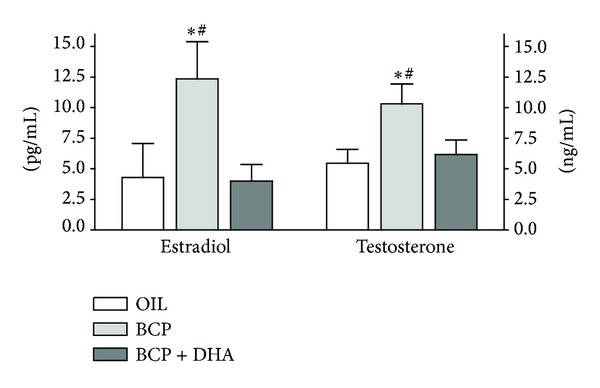
Testosterone and estradiol plasma levels determined at the end of the experimental sessions in OIL (white), BCP (light gray), and BCP+DHA (dark gray) groups. BCP administration for two weeks significantly increased E and T plasma levels, whereas BCP+DHA completely reversed this increase. Histograms represent means ± SEM. Estradiol: *P < 0.04 versus OIL and ^#^ *P* < 0.03 versus BCP+DHA. Testosterone: *P < 0.03 versus OIL and ^#^ *P* < 0.04 versus BCP+DHA.

*Estradiol (E) and Testosterone (T) Plasma Levels*. The effect of treatments on E and T plasma levels was examined at the end of the experiment. One-way ANOVA showed a significant effect of treatment on the E and T levels (*F*_2,14_ = 3.78, *P* = 0.048 and *F*_2,14_ = 3.76, *P* = 0.049, resp.). As shown in Figure 9, BCP treatment significantly increased E and T plasma levels (*P* < 0.04 and *P* < 0.026, resp.), while there were no changes in these hormones in the BCP+DHA group. In particular, T levels were 90% higher and E more than 150% higher in the BCP group than in the OIL and BCP+DHA groups.

## 4. Discussion

The main result of the present experiment is the strong analgesic effect recorded in animals treated with BCP and BCP+DHA in a model of persistent pain. Indeed, BCP alone or in combination with DHA resulted in a marked reduction of formalin-induced pain responses.

Chronic pain is a debilitating disease characterized most of the time by inflammatory processes underlying the main disease. This results in serious personal and social costs which pharmaceutical companies are addressing by producing synthetic or semisynthetic products. First-line pharmacological treatments for chronic pain include opioid and nonsteroidal anti-inflammatory drugs (NSAIDs), both of which are associated with serious side effects; for example, we have repeatedly found hypogonadism in association with opioid use [21, 22]. Therefore, there is the need for new pharmacological approaches that curtail the progression of the disease and reduce the pain but do not produce side effects. The “new” substances to be considered are mostly plant extracts, popularly known as natural analgesics. These compounds, essential oils including hundreds of elements or their extracts, offer great hope for the treatment of painful syndromes like fibromyalgia, low back pain, and musculoskeletal disorders, all of which present multiple symptoms often of multiple origin. Hence, it is imperative to carry out experiments able to clarify their effects. Another important aspect to be considered in the choice of these substances is that chronic pain is commonly associated with other medical conditions like depression, anxiety, insomnia, chronic fatigue, diabetes, cancer, and neurological dysfunctions, all requiring treatments that often add their side effects to those of the pain killers. These pathological aspects might also be significantly modulated by essential oil components.

In the present study, we have shown that *β*-caryophyllene (BCP) decreases pain with a long-term effect and without any apparent side effect. BCP, a common sesquiterpene present in cannabis and in many other plants, is a potent CB2 agonist producing peripheral antinociception [16, 23–25] without the central nervous system effects mediated by CB1 receptors [23, 26]. Therefore, the selective activation of CB2 receptors may produce peripheral pain relief without CNS psychotropic effects. BCP was effective in reducing neuropathic pain, in a CB2 receptor-dependent manner [27], and osteoporosis [5], and it prevented experimental colitis by reducing inflammation [28].

DHA belongs to the omega-3 family and has strong antioxidant and anti-inflammatory properties [29]. Our decision to use DHA instead of EPA was based on numerous studies suggesting that DHA is more useful than EPA. It has been shown that most of the neuroprotective effects of omega-3 oils are derived from the DHA component rather than the EPA component [30]. DHA also increases lipoxins, inhibits NFkb, and produces neuroprotectin-D and resolvins, which are important for complete resolution of the inflammatory process [31]; it is important to underline that the incomplete resolution of an inflammatory response can be a significant contributor to chronic and exacerbating disease and pain development.

Our* in vitro* acute toxicity tests indicated that BCP was toxic at high concentration, whereas its toxicity was completely reversed by concomitant DHA administration. Therefore,* in vivo* BCP analgesic activity was tested alone or in combination with DHA at half concentration. To model a clinical situation of persistent inflammation, we used repeated administration of a low concentration of formalin as irritant and BCP+DHA combinations were administered for two weeks. The formalin test was used because it allows pain intensity determination without any external interference. When injected in the paw, the irritant induces pain responses that can easily be quantified. In our laboratory, we determine each behaviour singly, that is, licking, flexing, or jerking of the injected paw [32], and thus it is possible to study the modulation of each neural circuit. Indeed, while paw jerk is mostly spinal cord mediated, licking and flexing are more complex responses involving longer subcortical reflex circuits. The general modulation of all three pain responses observed in the present study suggested general analgesia, probably due to attenuation of the peripheral inflammation. These results are in agreement with other studies reporting that BCP has analgesic and anti-inflammatory effects in both rats and mice [15, 16].

At present, we have no clear hypothesis concerning the interesting BCP+DHA synergistic activity. However, it is very likely that both substances show competitive binding/interaction for the same receptor since the modulation was also present in the* in vitro* experiment, in which the results cannot be explained by* systemic* effects.

Because of the endocrinopathies often observed in chronic pain patients, we collected blood samples at the end of the last formalin test to measure the testosterone (T) and estradiol (E) plasma levels in all experimental groups. These hormones were determined to study the possible modulatory effects of the tested substances upon the HPG axis or other related endocrine sites. The administration of BCP alone increased both the T and E plasma levels, while the combination of BCP at lower dose plus DHA did not change the hormonal plasma levels. Testosterone is* decreased* by pain as well as by pain killers [33], with a high number of sequelae that significantly decrease the quality of life in these patients. Our BCP+DHA treatment did not induce any decrease in the gonadal hormone levels. Moreover, in agreement with other experimental results by our group and others [32, 34], we have to consider the possibility that the analgesia could be testosterone-mediated. An interesting hypothesis is that higher levels of T and E may be involved in the observed analgesia via suppression of glial activity [35], since it has been shown that glial cells produce many inflammatory mediators leading to abnormal pain [36].

**Figure 10:**
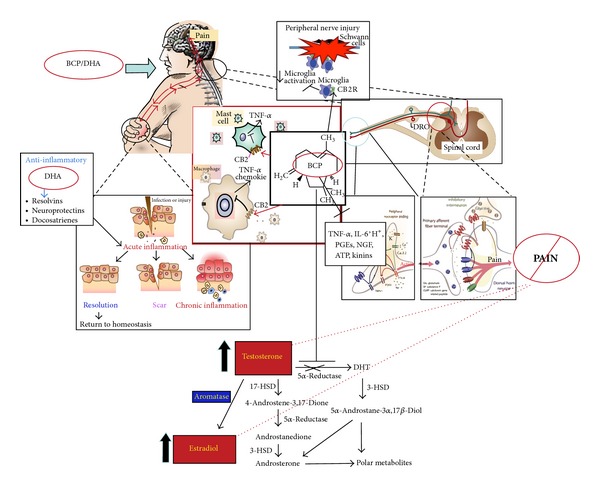
Failure of timely resolution of inflammation drives chronic inflammatory conditions and pain. Resolution of inflammation requires the elimination of key inflammatory cells and the downregulation of proinflammatory mediators at the inflamed sites. Docosahexaenoic acid (DHA) is converted into resolvins and protectins that counteract excessive inflammatory responses and stimulate proresolving mechanisms, tissue repair, and decreased pain. Beta-caryophyllene (BCP) reduces inflammation by its action on CB2 receptors, which are mostly expressed on immune cells and microglia.

In conclusion, the results of our experiment confirm the potent analgesic role of these compounds and their synergistic action and suggest their possible use in long-lasting treatments (Figure 10).
